# Delineating the Effects of Molecular and Colloidal Interactions of Dissolved Organic Matter on Titania Photocatalysis

**DOI:** 10.1021/acs.langmuir.2c03487

**Published:** 2023-02-06

**Authors:** Mostafa Maghsoodi, Céline Jacquin, Benoit Teychené, Geoffroy Lesage, Samuel D. Snow

**Affiliations:** † Department of Civil and Environmental Engineering, Louisiana State University, 3255 Patrick Taylor Hall, Baton Rouge, Louisiana 70803, United States; ‡ Eawag, Swiss Federal Institute of Aquatic Science and Technology, Überlandstrasse 133, 8600, Dübendorf, Switzerland; § IC2MP (Institut de Chimie des Milieux et Matériaux de Poitiers), UMR CNRS 7285), Université de Poitiers, 1 rue Marcel Doré, 86073 Poitiers, Cedex 9, France; ∥ IEM (Institut Européen des Membranes), UMR 5635 (CNRS-ENSCM-UM), Université de Montpellier, Place E. Bataillon, F- 34095 Montpellier, France

## Abstract

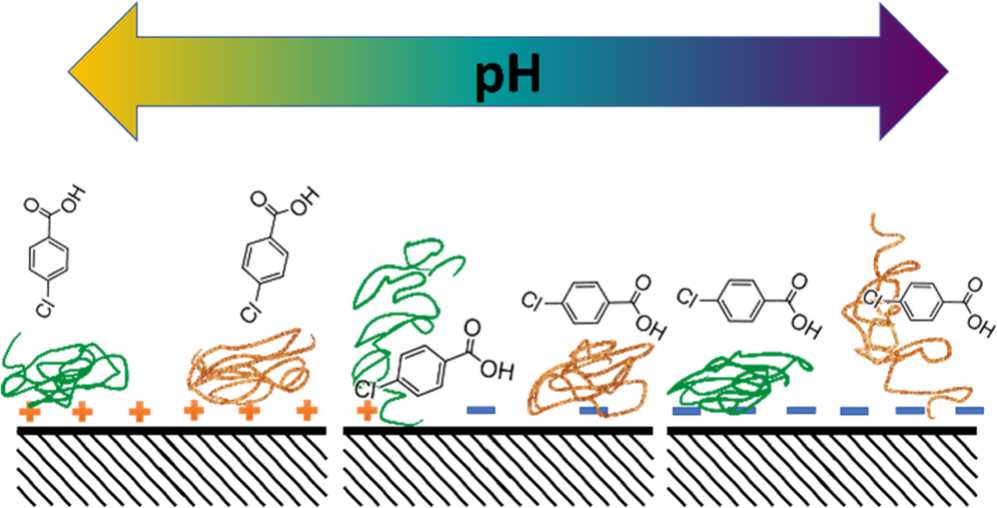

In the face of significant challenges to practical applications of photocatalysis for water treatment, recent reports revealed a potential route to overcome a problem posed by dissolved organic matter (DOM). These studies showed that inhibition of photocatalytic processes by DOM is driven largely by competition for active surface sites on TiO_2_ or other catalysts, and controlling the type of DOM present in solution could significantly mitigate DOM fouling. Whether or not control of solution parameters could achieve the same preventative action is not known. Here, a series of DOM isolates, including humic acid (HA) and transphilic (TPI), hydrophobic (HPO), or colloidal fractions of organic matter from a membrane bioreactor mixed liquor supernatant, were tested for inhibitory activity under a range of pH values (3, 5, 7, and 9) and ionic compositions (NaCl, CaCl_2_, and Al_2_(SO_4_)_3_ with ionic strengths (IS) ranging from 0 to 3 M). The resulting TiO_2_-DOM agglomerates were monitored for size and ζ-potential. Inhibitory profiles were generated using *para*-chlorobenzoic acid (*p*CBA) as probe with varying concentrations of inhibitory DOM for each solution condition to discern the extent of surface-phase quenching of radicals. Manipulation of pH clearly impacted inhibition, and the effect varied by DOM type; for example, interference occurred at all pHs for HA, at neutral or basic pHs for TPI, and only at pH 7 for HPO. Particle sizes did not correlate with inhibitory action of DOM. Increases in ionic strength induced growth of TiO_2_ and TiO_2_-DOM agglomerates, but again, particle sizes did not correlate to inhibition by DOM. The changes to IS, regardless of ion type, were not affected by the presence of TPI or HPO. Since particle stability did not correlate directly with photocatalytic activity, we suggest that surface-based quenching reactions arise from site-specific adsorption rather than generalized particle destabilization and aggregation.

## Introduction

Access to clean and safe drinking water is a critical challenge for people around the world, especially those who live in rural areas.^1,2^ Photocatalytic technologies may become a sustainable water treatment solution in some situations, if certain hurdles are overcome.^3,4^ The well-studied TiO_2_ offers an attractive option owing to its wide availability and high photocatalytic activity,^3,5−8^ but TiO_2_-based photocatalysis has yet to be utilized in practical water treatment systems.^7^ One of the primary barriers to commercialization is the mass transfer limitation of reactive oxygen species (ROS) to target contaminants,^5^ especially in complex water matrices.^9^ In fact, a 2019 study showed that less than 5% of generated ^•^OH radical in TiO_2_ photocatalysis is available for the degradation of target pollutants in natural waters due to interference of different solutes.^8^ Dissolved organic matter (DOM) is one of the most detrimental constituents because it inhibits photocatalytic processes competitively reacting with ROS and adsorbing to active surface sites for surface-phase reactions.^3,7,10^ Even in pure water, only a small portion of ROS produced by TiO_2_ diffuse away from the surface into the bulk solution,^11^ so the surface interactions between DOM and targeted pollutants are critically important. For successful application of photocatalysis, control over the type of DOM present in the process water is essential.

Several recent studies aimed to better understand the inhibitory mechanisms of DOM in photocatalytic systems. In 2015, Brame et al. experimentally validated an analytical model for accurately simulating photocatalytic performance of two different photoactive materials (TiO_2_ and Si-C_60_) when inhibited by either bulk or surface-phase scavenging; they concluded that the adsorption of DOM on the surface of the photocatalyst caused the most substantial reductions in performance; just 5 mg/L of Suwannee River natural organic matter was sufficient to reduce the TiO_2_-photodegradation rate for two probe molecules by half or more.^3^ They also noted that the adsorption affinity of DOM predicted inhibition when using a combined surface (Langmuir) and bulk inhibition model.^3^ This dynamic was later shown to be important in understanding the inhibitory effects of DOM in wastewater treatment;^12^ effluent organic matter from clean membrane decreased the photocatalytic efficiency by 100%, against 33% for the fouled membrane. This difference was explained by a higher permeation of colloidal DOM through the clean membrane, compared to the fouled membrane.^4^ In 2018, Luo et al. supplied an example of this phenomenon by showing that more humic acid (HA) adsorbed onto a TiO_2_ photocatalyst surface than fulvic acid (FA), due to higher hydrophobicity of HA, and consequently caused more inhibition.^13^ Similarly, we demonstrated that fractionated organic matter from activated sludge exhibited distinct inhibitory profiles, with the colloidal fraction causing more inhibition than transphilic (TPI) or hydrophobic (HPO) isolates.^4^

The progress in understanding photocatalyst quenching based on the nature of DOM is promising for addressing unwanted inhibitory reactions, but obvious questions remain regarding the roles of solution chemistry and cosolutes. At a basic level, simple colloidal physics could explain DOM’s inhibitory effects; for example, elevated ionic strength could induce aggregation of TiO_2_ particles with DOM via compression of the electrical double layer, which could increase surface quenching reactions. The situation may be more complex, however, when examined at the level of molecular-scale interactions. In a 2014 report, researchers showed that on positively charged surfaces, DOM (humic and fulvic acids) forms irreversibly sorbed, nanometer-scale adlayers.^14^ These DOM layers likely form on positively charged TiO_2_ below its isoelectric point, typically between 6 and 6.5.^7^ However, the impact of pH on DOM–TiO_2_ interactions is also complicated by the wide array of moieties constituting DOM, with numerous different p*K*_a_ values for the acidic groups.^15^ Alternately, DOM can serve as a stabilization agent for metal oxide nanoparticles via steric hindrance.^16,17^ In addition, ionic cosolutes may induce morphological changes in DOM; researchers have shown that the macromolecular structure of HA depended significantly on the type of cation in solution, with divalent cations exerting a stronger effect on size and conformation than monovalent cations.^18^ Little is known about how these ionic effects, whether particle stability or DOM conformational changes, impact photocatalysis in complex matrices. Control of intermolecular interaction dynamics may provide tools to manipulate the adsorption dynamics of DOM onto TiO_2_ by simple operational changes (e.g., pH adjustments or ion exchange processes) to mitigate surface-phase inhibition of photocatalysis.

The question of whether colloidal physics can predict the detrimental surface-phase quenching of photocatalysts by DOM remains outstanding. Here, particle stability experiments and inhibitory profiles of TiO_2_ photoactivity are used as tools to observe how changes to pH, ionic strength, or specific ionic constituents affect DOM–TiO_2_ adsorption and the consequent impacts on photocatalysis. Parameters are tested across colloidal stability thresholds to intentionally induce aggregation and co-precipitation of DOM and TiO_2_ in solution.

## Materials and Methods

### Chemicals

Humic acid, 4-chlorobenzoic acid (*p*CBA), and titanium dioxide (99.9% Anatase) with a nominal particle size of 32 nm and surface area of 45 m^2^ g^–1^ were obtained from Alfa Aesar (Haverhill, MA). Hydrochloric acid (HCl), sodium hydroxide (NaOH), magnesium sulfate (MgSO_4_), aluminum sulfate (Al_2_(SO_4_)_3_), sodium chloride (NaCl), and calcium chloride (CaCl_2_) were all purchased from VWR (Radnor, PA). A Nanopure Infinity system (Thermo Fisher Scientific, Inc., Waltham, MA) was used to produce ultrapure water (>18.2 MΩ cm). HPLC solvents including acetonitrile and phosphoric acid were purchased from Alfa Aesar, and all of them were of HPLC grade.

### Activated Sludge Organic Matter Collection

Three DOM fractions were used in this study including colloidal (C), transphilic (TPI), and hydrophobic (HPO) fractions. These isolates were obtained by processing the bulk supernatant samples collected from a full-scale membrane bioreactor wastewater treatment plant (La Grande Motte, France), as described previously.^19^ The DOM fractionation process was performed on the bulk supernatant obtained from a prefiltered activated sludge (1 μm) and concentrated with reverse osmosis membranes (TW30 Filmtech membranes). Then, the DOM fractions were isolated through dialysis bags (molecular weight cutoff, 3.5 kDa), XAD8 and XAD4 resins to collect colloidal, HPO, and TPI fractions, respectively.^4,20^ All fractions were finally freeze-dried for further use; thorough characterization of each has been performed previously.^19^

### Photochemical Experiments

Photodegradation experiments were performed in an enclosed UV cabinet equipped with a UV LED (LG Innotek UVC 6868, South Korea) with an emission peak at 278 nm (UV_278_) placed 20 cm from the reaction vessel, which was placed on a magnetic stirrer. To achieve quasi-collimated irradiation, a black tube was used to isolate rays from one of several lamps within the cabinet and mitigate reflection. An intensity value of 722 μW·cm^–2^ was measured for the UV LED using a BLUE–Wave UVNb-25 Spectrometer (StellarNet, Inc., Tampa, FL). The UV emission spectrum for the LED was reported previously.^21^ The vessel contained 10 mL solution, 5 mg L^–1^ TiO_2_ with 10 μM *p*CBA as a probe compound, which has a known reaction rate constant with ^•^OH.^22^ Inhibitory profiles were constructed by determining *p*CBA degradation rate constants across a series of DOM concentrations for each DOM sample (HA or the fractionated activated sludge isolates). Solution pHs were adjusted to 3, 5, 7, or 9 using HCl or NaOH when needed. The ionic strengths of solutions were adjusted from 0 to 3 M by adding salts with cations of varying valencies, NaCl, CaCl_2_, or Al_2_(SO_4_)_3_, when evaluating the effects of ionic strength or ion type on photodegradation rates. Samples were withdrawn at fixed time points for *p*CBA quantification via HPLC (Agilent Technologies, Inc., 1260 infinity). A C18 (125 mm) column was used in the analysis, and acetonitrile and 10 mM phosphoric acid were the mobile phase solvents (40:60) with a flow rate of 0.5 mL·min^–1^. The HPLC instrument had a variable-wavelength detector, which was adjusted to 234 nm for the detection of *p*CBA.^23^*p*CBA degradation rates were calculated for all of the experiments using linear regression in the plot of the natural log of *p*CBA concentration versus fluence, which followed first-order kinetics as demonstrated previously.^4,12^ Fluence values were calculated from intensity measurements according to Bolton and Linden,^24^ and each value accounted for reductant transmission factors: Petri, water, divergence, and reflection.

### Aggregate Characterization

The hydrodynamic diameter and ζ-potential of pristine TiO_2_ and (presumed) TiO_2_-DOM aggregates were measured using dynamic light scattering (DLS) and phase analysis light scattering (PALS) using a Malvern Zetasizer Nano ZS90 (Malvern Instruments, Worcestershire, U.K.). TiO_2_ solutions were sonicated for 5 min before adding to a given solution and mixed with an experimental solution for 2 min before taking DLS or PALS measurements. PALS measurements were performed in a folded capillary cell. A refractive index of 2.524 was assigned to TiO_2_ nanoparticles.^25^ Error values for size and ζ-potential represent standard errors computed by the Malvern Zetasizer Software, based on a minimum of 10 measurement runs per sample and two samples per datum. Aggregate size and ζ-potential measurements were compared across experimental conditions using three-factor ANOVA tests using SigmaPlot v. 14.0 software (Systat Software, Inc., San Jose, CA) with significance defined as *p*-value < 0.05. Three ANOVA tests were performed: one for the size of particles based on changes in DOM concentration, DOM type, and pH; second under the same categories for ζ-potential; and third for the size of particles with changing IS, ion type, and DOM types.

## Results and Discussion

### Impacts of DOM and pH on Particle Size and ζ-Potential Variations

**Figure 1:**
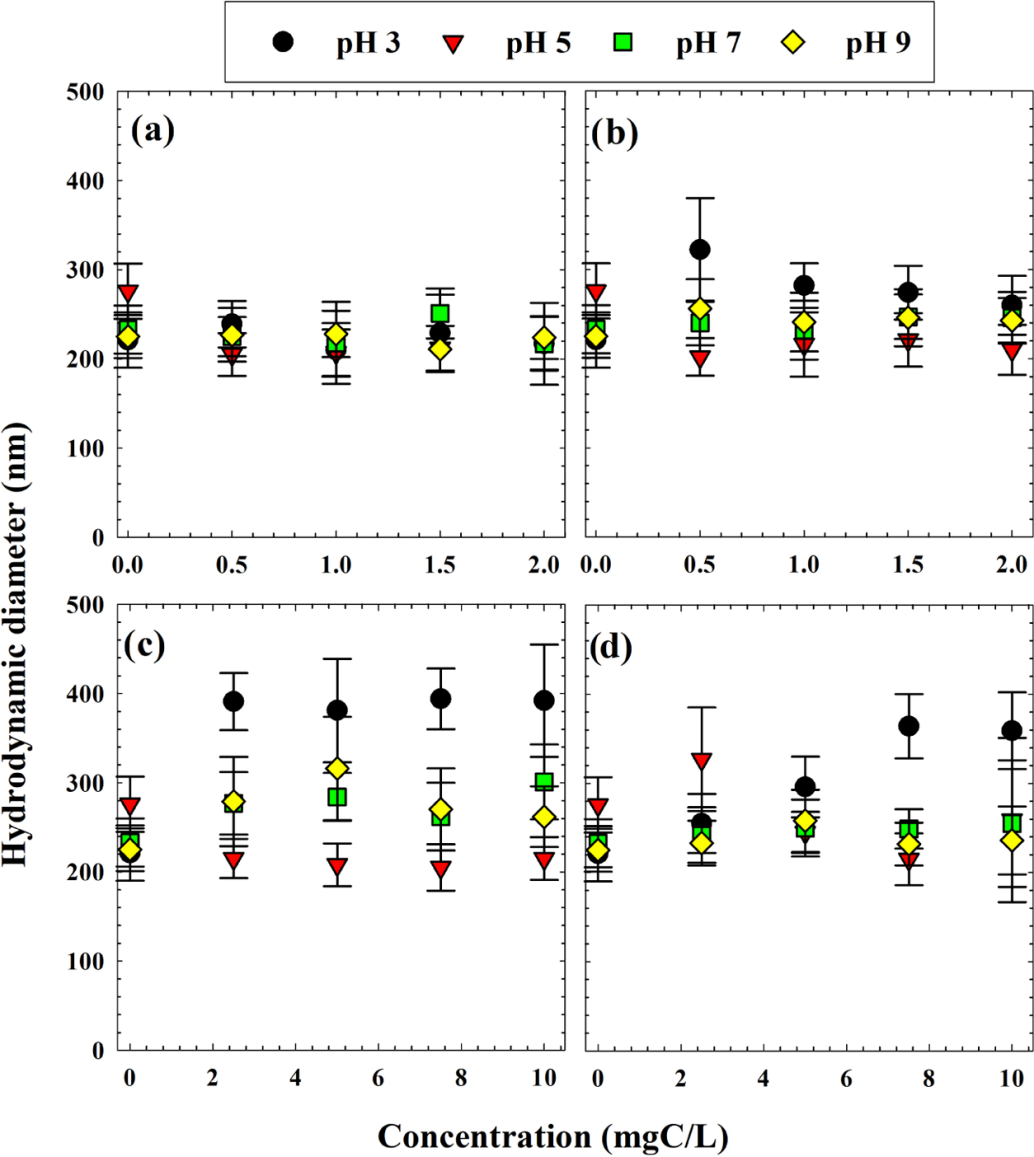
Hydrodynamic diameters of TiO_2_ particles at pH 3, 5, 7, and 9 in the presence of (a), HA (b), colloids (c), TPI, and (d) HPO. Error bars indicate standard error.

To determine the role of pH in the aggregation of TiO_2_ in the absence or presence of DOM fractions, the hydrodynamic diameter and ζ-potential of TiO_2_ were measured at various pHs. Particle ζ-potential measurements were conducted across DOM concentrations found to be meaningful to photocatalytic quenching in our previous work, up to 2.0 mgC/L for HA and C or 10 mgC/L for TPI and HPO (these concentrations were categorized as lowest, low, high, and highest for ANOVA purposes).^4^ Particle size data are shown in Figure 1 for each DOM isolate across pH values. In general, hydrodynamic diameters varied little with DOM concentration; mean sizes are recorded in Table S1, with corresponding ANOVA results shown in Tables S2–S4. DOM type and pH were both found to significantly (*p*-value < 0.05) alter aggregate size, while DOM concentration did not (Table S2). Pairwise comparisons between factor groups revealed (Table S3) that particle sizes for HA differed significantly from both TPI and HPO but not from colloids. TPI was also found to be statistically different compared to colloids. At pH 3, TiO_2_-DOM particle size differences were significant (>40 nm differences in means), but no significant difference was found for any other pH pairing (<12 nm differences in means).

**Figure 2:**
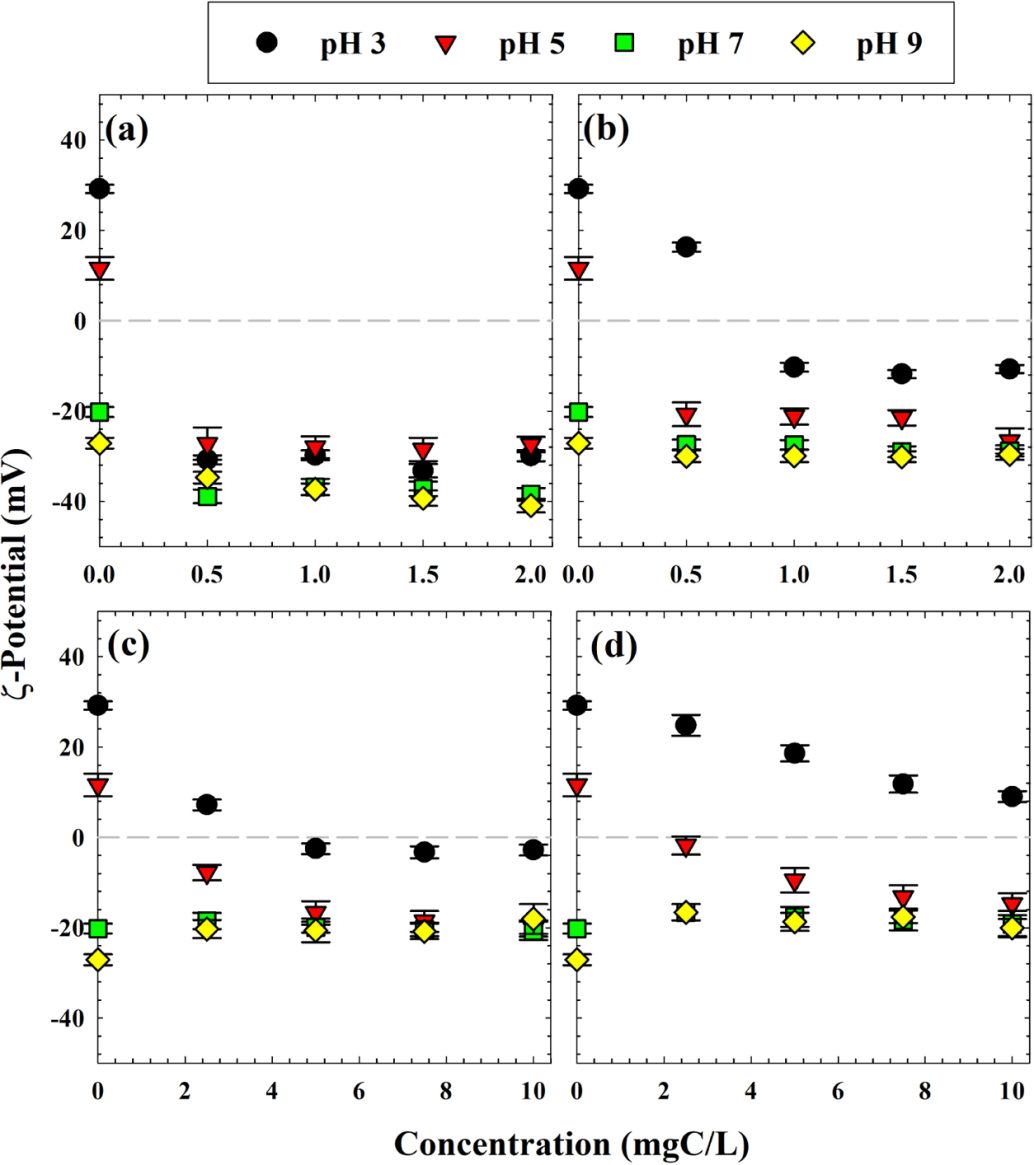
TiO_2_ particle ζ -potential values as a function of DOM concentration at pH 3, 5, 7, and 9 for (a) HA, (b) colloids, (c) TPI, and (d) HPO. Error bars indicate standard error.

Particle sizes (Figure 1) were generally inversely correlated with ζ-potential magnitudes, which are plotted in Figure 2 and enumerated in Table S5. Corresponding ANOVA results are shown in Tables S6–S8. The isoelectric point of colloidal TiO_2_ is typically between pH 6.0 and 6.5,^7^ so the proximal pH cases (5 and 7) without DOM had smaller absolute ζ-potential values (+11.6 and −20.2 mV, respectively) than pH 3 (+29.2 mV) or pH 9 (−27.1 mV). Three-factor ANOVA analysis revealed that each of the three factors (pH, DOM type, and DOM concentration) affected the particle ζ-potential significantly (Table S6). HA yielded surface ζ-potentials significantly more negative than each of the other DOM isolates (Table S8), according to pairwise comparisons (Table S7). In order of negativity of ζ-potentials across the different DOM concentrations and pH values, HA was most negative, followed by colloids, TPI, and then HPO. The presence of DOM, compared to the zero-DOM case, caused significant changes to ζ-potential no matter the DOM concentration, but no differences were statistically different when comparing different levels of DOM content. This observation suggests that small amounts of DOM, relative to impacts on photocatalytic outcomes,^4^ exerted meaningful changes to particle surface characteristics. Adjustments to solution pH were also found to significantly impact ζ-potential across each pairing, except for pH 7 with pH 9 (Table S7).

Several trends within individual experimental factors revealed notable trends in particle ζ-potentials. The addition of HA at pH 5 changed the ζ-potential from a low absolute value of +11.6 ± 2.5 mV to a higher, negative value of −27.8 ± 3 mV (average value calculated within the DOM concentration range, Table S5). The HA also decreased TiO_2_ particle size from 276 ± 31 to 211 ± 26 nm. With positively charged TiO_2_ at pH 5, the HA likely forms a water-rich, highly polar adlayer, as documented for a system of HA adsorbing onto positively charged, amine-terminated self-assembling monolayers by Armanious et al.;^14^ this dynamic could stabilize the TiO_2_ by expressing negatively charged moieties out into solution, thereby explaining the reduced particle sizes. This HA-induced particle size reduction occurred at all other pH values tested, where the ζ-potential became more negative. Notably, the changes in ζ-potential provide good evidence for significant HA adsorption, while decreasing particle sizes show that a stabilization effect precludes conclusions on the basis of particle size only. This observation is in line with a study by Jayalath et al., which noted that HA-adsorbed TiO_2_ had a higher magnitude of ζ-potential than bare TiO_2_, regardless of HA concentration, resulting in greater particle stability in all cases.^10^ Modification of surface properties by adsorption of negatively charged DOM also occurs at low pH values; most DOM molecules contain numerous carboxylic functional groups, which provide wide acid/base equilibria ranges at around pH 3-4.^26−28^ In our case, the fractionated bulk supernatant samples yielded successively less negative particles at pH 3—where TiO_2_ is normally positively charged—in order of C <TPI <HPO, and this trend matched well with reported carboxylic acid functionality in naturally derived DOM isolates, where an HPO fraction had significantly less carboxylic content than a TPI counterpart (colloidal matter were not studied in that report).^28^ With fewer carboxylic groups, HPO is consequently less prone to electrostatic adsorption to the hydrophilic, positively charged TiO_2_. At pH 5, the addition of colloids increased the magnitude of ζ-potential to a lesser extent than HA, but particle sizes still decreased, showing that the colloidal fraction exerted a stabilization effect similar to HA at pH 5. Here again we note that colloids likely form a highly polar, stabilizing adlayer on the surface of TiO_2_.^14^ A decrease in particle sizes was also observed for the TPI case at pH 5, but no change was observed in the size in the case of HPO. The TPI fraction did not follow the expected ζ-potential-size stabilization trend at basic pHs. TiO_2_ particle sizes increased from 211 (±24) at pH 5 to averages of 281 (±36) or 282 (±47) nm at pHs 7 or 9, respectively, despite higher ζ-potential magnitudes. Overall, particle ζ-potentials were generally inversely correlated to sizes, as expected. Notably, the different DOM samples had varying charge neutralization capacities; at pH 3, DOM imparted negative charge in order of HA > colloids > TPI > HPO in terms of magnitude of charge per concentration.

### Impacts of pH-Driven DOM–TiO_2_ Interactions on Photocatalysis

**Figure 3:**
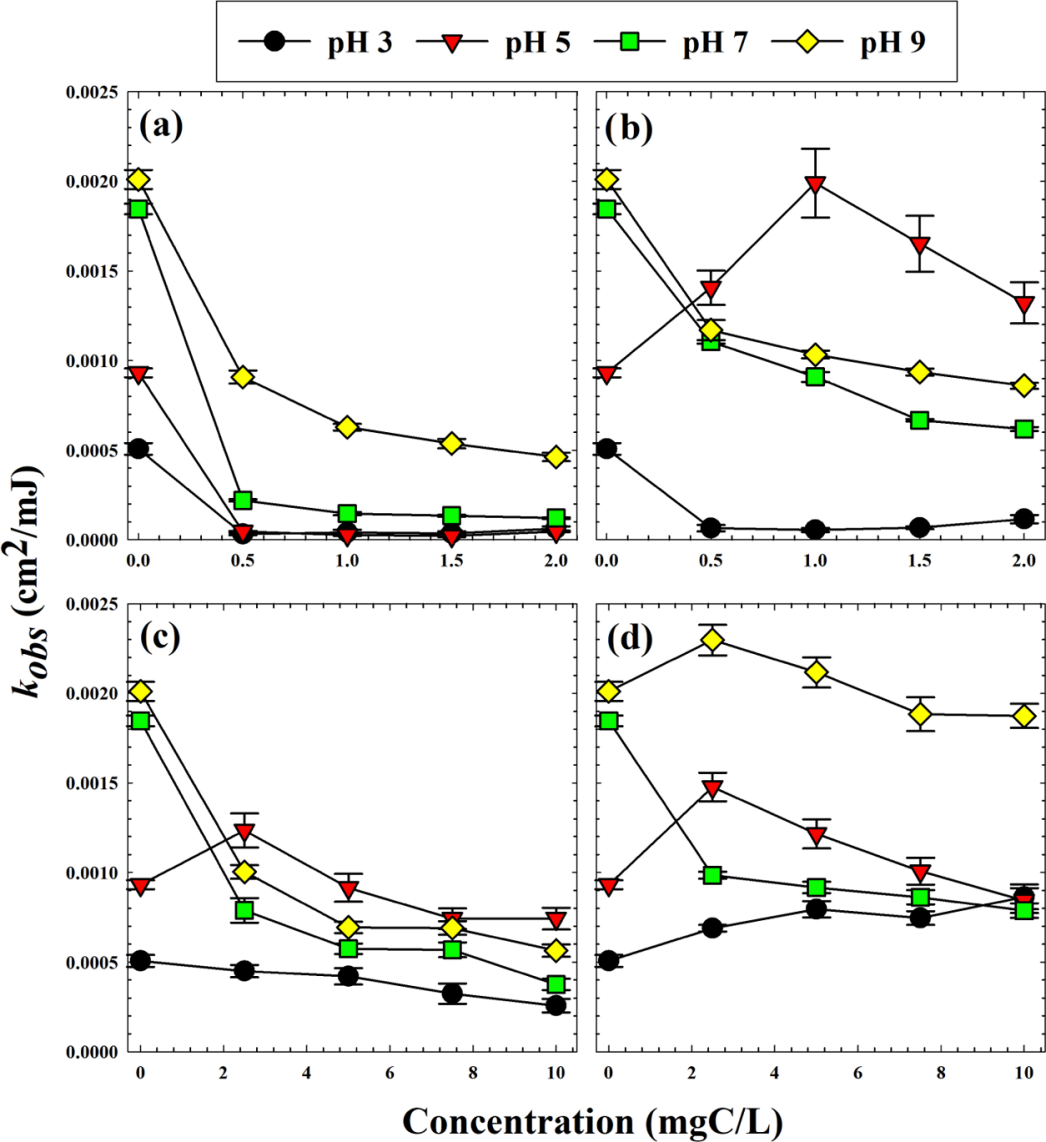
Observed *p*CBA degradation rate constants (first order with respect to fluence) in the presence of 5 mg/L TiO_2_ and various DOM concentrations at pH 3, 5, 7, and 9 for (a) HA, (b) colloids, (c) TPI, and (d) HPO. Error bars indicate standard error of linear trendlines with 95% confidence.

A series of *p*CBA photodegradation experiments were conducted in the presence or absence of DOM samples, as shown in Figure 3. Data used to generate these rate constants are shown in Figure S1. The lowest photodegradation rate constant (highest inhibition by DOM when present) occurred at pH 3 for nearly all cases. This observation was somewhat surprising since ^•^OH radical production is known to increase at low pHs via reactions between electron holes in the TiO_2_ surface with protons in solution.^29^ The reason for the reduced rate is likely related to limited adsorption of *p*CBA compound onto the TiO_2_ surface. At pH 3, deprotonation occurs for only about 10% of *p*CBA molecules since its p*K*_a_ is 3.98.^30^ Consequently, *p*CBA is less likely to adsorb onto the TiO_2_ surface, since most of the molecules are not electrostatically attracted to the positively charged TiO_2_.^31^ Because ^•^OH are generated at the TiO_2_ surface and only a small portion of them diffuse away,^11^ the adsorption of *p*CBA on the surface is a critical factor in its degradation rate. The low photodegradation constants at pH 3 could also be explained by competitive adsorption of Cl^–^ (added via HCl) for adsorption sites on TiO_2_ surface. Piscopo et al. noted that chloride competes with target compounds (*p*CBA in this case) for adsorption sites, resulting in reduced in photodegradation rates.^32^ The decrease in the rate constants for *p*CBA degradation here indicates the adsorption of target contaminant onto the photocatalyst surface is a rate-controlling factor regardless of the concentration of generated ^•^OH. The addition of HA completely inhibited the photodegradation. Colloids also showed strong inhibition of photocatalysis at pH 3, and their nonlinear inhibitory profile of colloids indicated strong affinity toward TiO_2_. HA exhibits a net negative charge in all ranges of pH used here,^33^ and similar to the colloids, HA adsorbs strongly onto positively charged TiO_2_ surfaces under acidic conditions. For both HA and the colloidal DOM, surface-phase inhibition was the dominant quenching mechanism at pH 3, whereas TPI presented a profile representative of bulk quenching.^3^

Given the different pathways of DOM interference, the inhibitory effect of TPI at pH 3 was milder compared to HA or colloids where the degradation rate in the presence of TPI was 4.5 × 10^–4^ cm^–2^·mJ at a concentration of 2.5 mgC/L, much higher than the same in the presence of HA or colloids at a concentration of 2.0 mgC/L (∼1.10^–4^ cm^–2^·mJ). A comparatively low affinity between TiO_2_ and TPI could be inferred by this observation, but the opposite was expected according to the analysis of the average hydrodynamic diameters of TiO_2_, which indicated increased aggregation under these conditions. Although the average hydrodynamic diameter of TiO_2_ increased with the addition of TPI (390 +/– 47 nm) compared to HA (224 +/– 32 nm) or colloids (285 +/– 37 nm), the resultant inhibition profiles were strikingly different, suggesting that the aggregation of TiO_2_ nanoparticles is not the key factor in the inhibition of photocatalysis. Destabilization of TiO_2_ particles via DOM addition does not imply direct competition for photocatalytic surface sites.

HPO added to the TiO_2_ system improved its photocatalytic performance at pH 3, counter to the expectation that nearly any DOM will compete with *p*CBA to scavenge ^•^OH in the bulk phase if not on the TiO_2_ surface. This surprising enhancement to degradation kinetics suggests that HPO at pH 3 acts to accelerate *p*CBA oxidation in some way. The role of photochemically produced reactive intermediates is important in some DOM systems, especially where singlet oxygen is sensitized by DOM.^34^ However, in our study, the kinetic enhancement effect is pH-dependent, which suggests that surface phenomena (e.g., favorable co-adsorption processes) are a more likely explanation. HPO also improved *p*CBA photodegradation kinetics at pH values 3, 5, and 9 but appeared to cause mild inhibition via surface and bulk competition at pH 7.^3^ A kinetic enhancement was also observed for the colloidal DOM at pH 5, in stark contrast to its strong, surface competition inhibition at other pHs. With the exception of HA, this enhancement effect was also observed for all DOM samples at pH 5; the increases occurred at low DOM/TiO_2_ ratios, typically followed by up decreases with higher DOM concentrations. For example, the addition of up to 1 mgC/L colloids at pH 5 improved the photodegradation kinetics, at which point the trend reversed, suggesting that inhibitory mechanisms began to counterbalance the improvements. For TPI and HPO, increasing the concentration beyond 2.5 mgC/L caused inhibition. Drosos et al. noted a similar improvement at low ratios of DOM/TiO_2_ and concluded that the enhancement is caused by a reduction in diffusion limitations when DOM attracts targeted contaminants to the surface of TiO_2_.^35^ They also showed that at high DOM:TiO_2_ ratios, DOM blocks the target contaminants from reaching the surface of TiO_2_.^35^

At pH 7, all DOM types inhibited *p*CBA photodegradation, with HA exerting the strongest effect. The inhibition by HA and colloids was likely caused by strong adsorption interactions, given the exponential decrease of rates with increasing DOM, while HPO and TPI appeared to inhibit partly via surface and partly by bulk quenching. Photodegradation experiments at pH 9 showed largely the same results, with the notable exception of HPO, which demonstrated an enhancement effect similar to HPO at pH 3 or pH 5. At neutral and basic conditions, TiO_2_ and *p*CBA both have negative charges and experience some electrostatic repulsion, and poor *p*CBA-TiO_2_ adsorption at high pHs was reported by Jayalath and co-workers in 2018, where they showed a decreasing trend in the adsorption of HA with the increase in pH.^10^ On the other hand, large DOM molecules with varied functional groups and charge distributions are more likely to sorb partly, if not wholly, onto TiO_2_. Indeed, partial adsorption of DOM molecules is precisely the mechanism we suppose is responsible for the photodegradation enhancement observed in some cases, and the HPO fraction is the least likely to have fully favorable interactions with hydrophilic TiO_2_. Note that TiO_2_ is least charged, therefore most hydrophobic, at pH 7, which is where HPO competes most strongly with *p*CBA for adsorption sites.

### Impacts of IS-Driven DOM–TiO_2_ Interactions on Photocatalysis

The effect of ionic strength on the aggregation of TiO_2_ was investigated by adding different salts to solutions in the presence or absence of different fractions, and the pH of solutions was adjusted to 6.4. The TPI and HPO fractions were selected for comparison because linear DOM-inhibitory profiles were observed for these fractions under dilute conditions in our previous work,^4^ implying that the inhibition was primarily bulk-phase competition.^3^ These fractions, then, provide an excellent test case to determine whether particle destabilization will induce surface-phase inhibition or if the presence of multivalent ions will otherwise affect the system.

**Figure 4:**
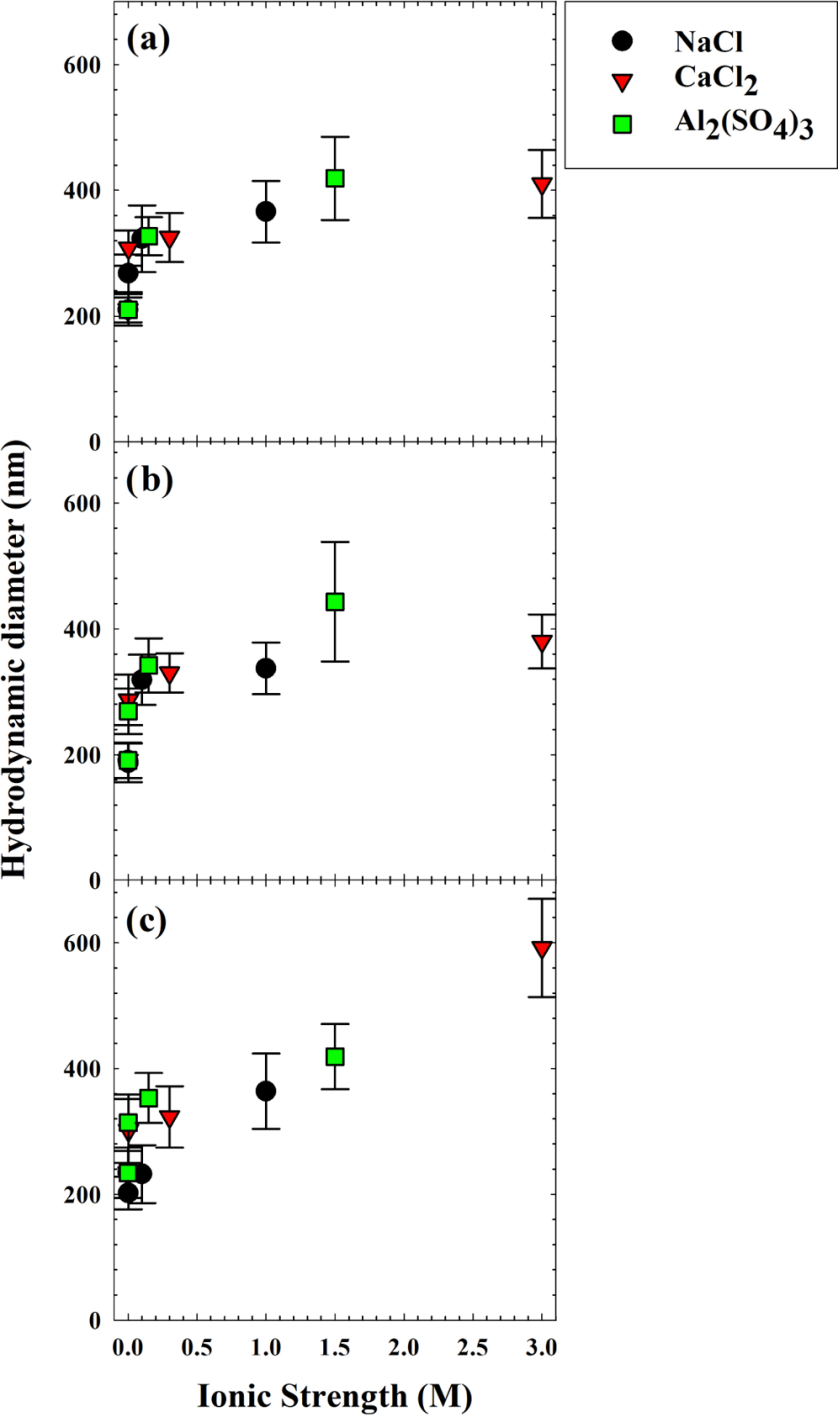
Hydrodynamic diameters of TiO_2_ particles as a function of IS (pH 6.4) added via NaCl, CaCl_2_, or Al_2_(SO_4_)_3_ for cases (a) without DOM, (b) with 10 mgC/L TPI, and (c) with 10 mgC/L HPO. Error bars indicate standard error.

DLS measurements were collected for TiO_2_ particles under a variety of cosolute conditions; these data are plotted in Figure 4 and shown in Table S9. Corresponding ANOVA results are shown in Tables S10–S12. With a fixed pH value, surface charge characteristics should not change meaningfully in direction or magnitude with changing IS; the electrical double-layer distance should compress with increasing IS,^36,37^ but the PALS platform was not suitable to discern these changes in terms of ζ-potential. The three-factor ANOVA found that only IS impacted particle diameters significantly across the conditions tested (Table S10); the TiO_2_ particles aggregated more with increasing ionic strength (Table S12). The particle diameters in these solutions were largely unaffected upon further addition of HPO or TPI, with two exceptions. When CaCl_2_ was added to yield an ionic strength of 3 M, larger aggregates formed with HPO compared to the TPI and no-DOM cases. Likewise, when Al_2_(SO_4_)_3_ was added to an ionic strength of 1.5 M, a small particle size increase was observed with HPO. These observations suggest first that the TPI and HPO fractions generally do not stabilize or destabilize TiO_2_ particles with increasing ionic strength and second that the HPO molecules may be affected differently by multivalent cations, enhancing TiO_2_ aggregation in the presence of Ca^2+^ with Cl^–^ (3 M ionic strength) or of Al^3+^ with SO_4_^2–^ (1.5 M ionic strength). Note that this amount of aluminum sulfate is expected to induce sweep coagulation with the formation of Al(OH)_3_ solids, kept in suspension via stirring. The extreme condition here provides a case of maximum particle destabilization. While 3 M ionic strength may not be relevant in real systems since typical seawater has an ionic strength of about 0.7 M,^38^ the results suggest that conformational changes in DOM do not necessitate changes in TiO_2_ agglomeration. For instance, millimolar concentrations of CaCl_2_ were found to cause significant changes in size and conformational to humic substances by Baalousha and co-workers.^18^

### Effects of IS and DOM on Photocatalysis

**Figure 5:**
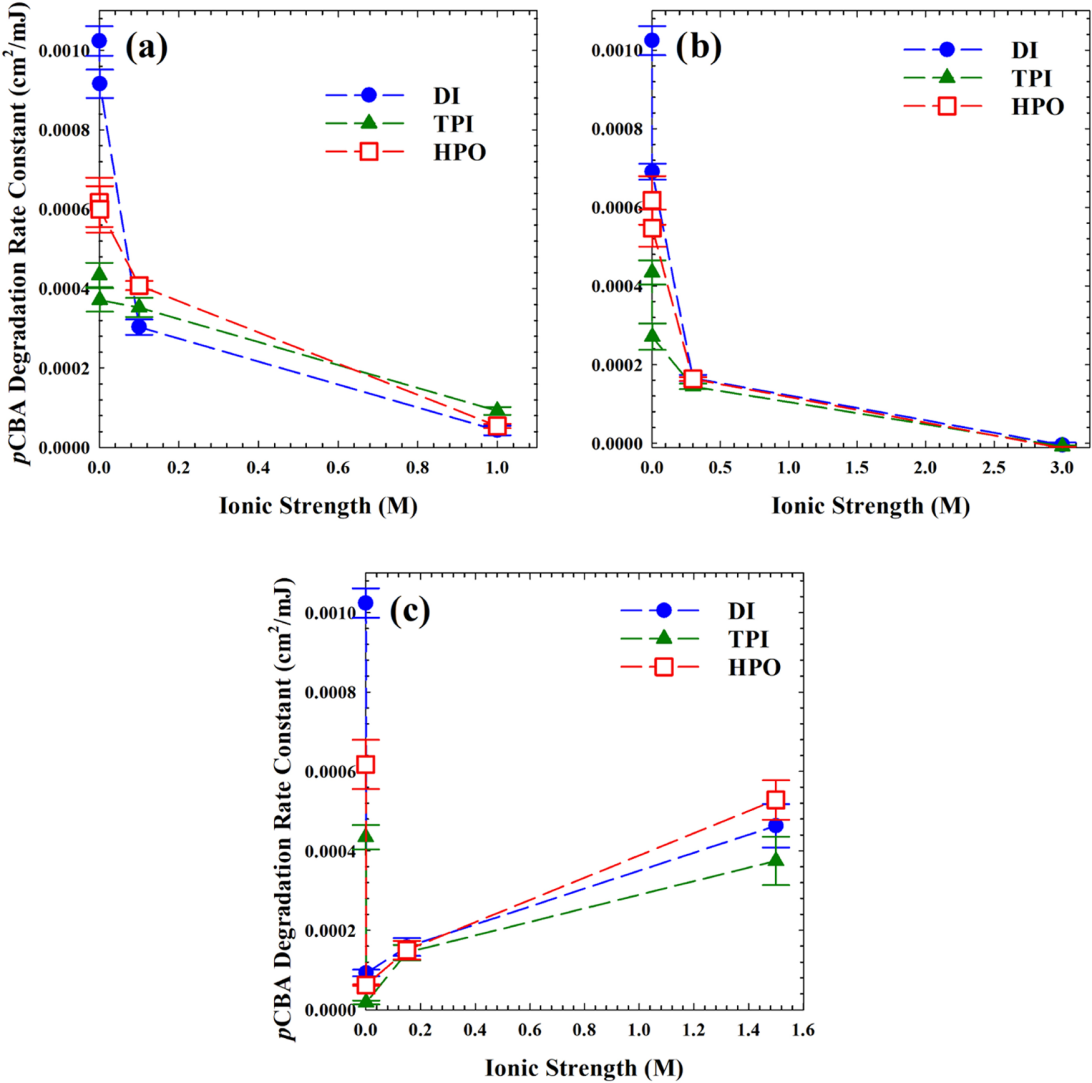
*p*CBA degradation rate constants in the presence of 5 mg/L TiO_2_, TPI, and HPO in various ionic strengths of (a) NaCl, (b) CaCl_2_, and (c) Al_2_(SO_4_)_3_. Error bars indicate standard error of linear trendlines with 95% confidence.

Photodegradation experiments were also performed across ranges of IS and DOM concentrations to determine if destabilization of DOM–TiO_2_ systems induces inhibition via surface competition. Results from these experiments are arrayed in Figure 5. Data used to generate these rate constants are shown in Figure S2. The increase in IS decreased the photodegradation rates in all cases, even with only a small increase in IS (0.01–0.03 M). Generally, decreases in photoactivity may be expected with increasing IS due to compression of the electrical double layer and consequent agglomeration of TiO_2_ with itself or DOM. However, our scrutiny of changes in agglomeration due to pH demonstrated that particle size may not be a reliable predictor of photoactivity. Although reaction rates do depend on the physical properties of TiO_2_ nanoparticles, including size,^39^ Lin et al. noted that there are disagreements in this area; some researchers claim that the efficiency of photocatalytic processes did not increase monotonically with a decrease in particle size.^40^ According to Dionysiou et al., increasing IS decreased *p*CBA adsorption onto TiO_2_ surfaces, reducing the photodegradation rate in their system.^41^ Reductions in *p*CBA adsorption onto TiO_2_ is a more likely explanation of the decrease in *p*CBA degradation rates observed here. Researchers have shown that chloride in particular competes with some aromatic compounds for surface adsorption sites.^32^

The addition of different salts in small amounts (0.01–0.03 M as IS) caused a range of inhibition according to the following order: Al_2_(SO_4_)_3_**>** CaCl_2_**>** NaCl. Recent work by Raza et al. (2016) demonstrated that electrolyte type plays an important role in the morphology of TiO_2_–DOM aggregation: Cl^–^ induced tight aggregates, while NO_3_^–^ promoted loose aggregation with larger particle sizes.^42^ The observed ordering of inhibition here suggests that morphological changes associated with the valency of ions in solution may explain the differential photocatalytic performances. Moreover, Wang et al. also confirmed that Ca^2+^ and other divalent cations caused more inhibition than Na^+^.^43^ Increasing the IS lowered the observed reaction rate constants in both NaCl and CaCl_2_ cases, but Al_2_(SO_4_)_3_ accelerated the reaction after the initial decline. This effect was also observed by Wang et al.; they postulated that the mechanism was a hydrolytic effect by Al^3+^, where poly-aluminum hydroxides form by reactions of Al^3+^ with H_2_O, releasing some H^+^.^43^ Notably, this effect did not occur in a low concentration of Al_2_(SO_4_)_3_ as the results showed almost complete inhibition by increasing the IS with Al_2_(SO_4_)_3_ to 0.015 M.

The effects of ionic species on DOM–TiO_2_ interactions were examined using TPI and HPO. Since both TPI and HPO exhibit bulk phase inhibition in dilute solutions,^4^ ion-induced adsorption of the DOM onto TiO_2_ should be apparent. The decreases in photodegradation rate constants caused by increasing IS were smaller for TPI and HPO compared to DI. The addition of 0.015 M Al_2_(SO_4_)_3_ caused a decrease of 0.0009 cm^–2^·mJ for the rate constant in DI, which was about twice the change observed with TPI (0.0004 cm^–2^·mJ) or HPO (0.0005 cm^–2^·mJ) in solution. This observation was surprising because DOM is a major source of quenching for ^•^OH and adsorption of the DOM onto TiO_2_ was expected to increase with IS due to particle destabilization. The resulting rate constants, however, were clustered near a floor value of ∼0.0001 cm^–2^·mJ with minimal photoactivity retained. In the NaCl and CaCl_2_ cases, the added salts appeared to cause the same suppression of photoactivity at all IS values, regardless of the presence of TPI or HPO, since the DI case tracked closely with the TPI and HPO trends. Manipulation of IS did not meaningfully alter the surface competition dynamics (or lack thereof) of TPI or HPO onto TiO_2_ particles.

## Conclusions

In the search for applied, practical photocatalytic water treatment, recent work revealed an important parameter for designing effective systems; adsorption interactions of target and interfering molecules onto catalyst surfaces can be used as a tool to optimize photocatalysis in complex waters.^3,4^ Here, the DOM–TiO_2_ surface adsorption mechanism was scrutinized by inducing adsorption and particle aggregation via changes in pH and IS across conditions expected to completely destabilize the DOM–TiO_2_ colloidal particles. Resulting photocatalytic performances highlighted several critical points for controlling the unwanted DOM quenching reactions in photocatalytic systems:

- Adjustments to pH induced aggregation near TiO_2_’s isoelectric point, but observed increases in aggregate size did not correlate to increased inhibition, contrary to prior reports.^44,45^
- Particle ζ-potential predicted aggregate sizes but did not directly correlate with inhibition. The DOM types which imparted more negative surface charge at pH 3 were the stronger inhibitors, in order of HA, colloids, TPI, and HPO.
- Increases to IS reduced photocatalytic performance in most cases, and aggregate sizes increased with increasing IS. But aggregate sizes were not predictive of inhibition for the ionic conditions tested.
- The effect of IS on photocatalytic performance was unchanged by the presence of TPI or HPO regardless of salt type used.

The simple fact that DOM–TiO_2_ agglomerate size cannot predict photocatalytic inhibition has profound implications on prospective photocatalytic applications and quenching mitigation strategies. Neither the compression of the electrical double layer nor DOM conformational changes by multivalent cations were important in the DOM–TiO_2_ system. Site-specific interactions between DOM molecules and the photocatalyst surface are fundamental to the inhibitory process, as demonstrated by the stark differences between inhibition profiles of the DOM isolates studied here at different pHs. In fact, DOM can promote photodegradation of target molecules at rates beyond pure water cases under certain circumstances; this phenomenon appears to be directly related to the dynamics of molecular interactions between the target (*p*CBA here), DOM molecules, and the TiO_2_ surface. Further investigations on the fundamental interactions of moieties of target and inhibitory compounds will be fruitful for efforts aimed at predicting and preventing inhibition of photocatalysts by non-target organics.

## Supporting Information Available

Tables of DLS and PALS data and three-way ANOVA statistics for TiO_2_ particle sizes and ζ-potential with variable solution conditions, and figures of *p*CBA photodegradation profiles according to pH, DOM type, IS, and ion species (PDF)
