# Supplementary material for: Delineating the Effects of Molecular and Colloidal Interactions of Dissolved Organic Matter on Titania Photocatalysis
Source: Langmuir. 2023 Feb 6;39(10):3752–61. doi: 10.1021/acs.langmuir.2c03487 (PMC10018764; doi:10.1021/acs.langmuir.2c03487)
Supplement: Supplementary file 1 — la2c03487_si_001.pdf [file la2c03487_si_001.pdf]

Supporting Information

Title: Delineating the effects of molecular and colloidal interactions of dissolved organic matter on titania photocatalysis

Authors: Mostafa Maghsoodi[1], Céline Jacquin[2], Benoit Teychené[3], Geoffroy Lesage[4], Samuel D. Snow[1*]

[1]Department of Civil and Environmental Engineering, Louisiana State University, 3255 Patrick Taylor Hall, Baton Rouge, Louisiana 70803, United States.
[2]Eawag, Swiss Federal Institute of Aquatic Science and Technology, Überlandstrasse 133, 8600, Dübendorf, Switzerland
[3]IC2MP (Institut de Chimie des Milieux et Matériaux de Poitiers), UMR CNRS 7285), Université de Poitiers, 1 rue Marcel Doré, 86073 Poitiers Cedex 9, France
[4]IEM (Institut Européen des Membranes), UMR 5635 (CNRS-ENSCM-UM), Université de Montpellier, Place E. Bataillon, F- 34095, Montpellier, France.
*Corresponding author: ssnow@lsu.edu;

Contents
Number of pages: 10
Tables: 12
Figures: 2

Table S1. Mean hydrodynamic diameter and standard error for TiO_2 in the absence and presence of DOM at various pH values.

Constituents	pH 3	pH 5	pH 7	pH 9
	Particle size (nm)			
**TiO_2**	221 (±31)	276 (±31)	233 (±27)	225 (±24)
**TiO_2 and HA**	224 (±32)	211 (±26)	228 (±36)	223 (±27)
**TiO_2 and Colloids**	285 (±37)	212 (±29)	241(±28)	247 (±31)
**TiO_2 and TPI**	390 (±47)	211 (±24)	281 (±36)	282 (±47)
**TiO_2 and HPO**	319 (±37)	262 (±51)	249 (±39)	240 (±31)

Table S2. Three-factor ANOVA results for TiO_2-DOM particle hydrodynamic diameters with changing pH, DOM type, and DOM concentration.

**Source of Variation**	**DF**	**SS**	**MS**	**F**	***p-value***
**DOM Type**	3	33870.737	11290.246	8.094	<0.001
**DOM Conc**	4	4098.95	1024.738	0.735	0.571
**pH**	3	33632.638	11210.879	8.037	<0.001
**Residual**	69	96251.063	1394.943	-	-
**Total**	79	167853.39	2124.726	-	-

Table S3. Pairwise multiple comparison results for TiO_2-DOM hydrodynamic diameters using the Holm-Šidák method, given differing pH, DOM concentration, and DOM type.

**Comparison**	**Diff. of Means**	**t**	**P**	**P<0.050**
**Comparison factor: DOM Type**				
**TPI vs. HA**	55.7	4.716	<0.001	Yes
**HPO vs. HA**	36.85	3.12	0.013	Yes
**TPI vs. Colloids**	35.7	3.023	0.014	Yes
**Colloids vs. HA**	20	1.693	0.259	No
**TPI vs. HPO**	18.85	1.596	0.217	No
**HPO vs. Colloids**	16.85	1.427	0.158	No
**Comparison factor: pH**				
**3 vs. 5**	53.25	4.509	<0.001	Yes
**3 vs. 9**	44.35	3.755	0.002	Yes
**3 vs. 7**	41.05	3.476	0.004	Yes
**7 vs. 5**	12.2	1.033	0.665	No
**9 vs. 5**	8.9	0.754	0.702	No
**7 vs. 9**	3.3	0.279	0.781	No

Table S4. Least square means for TiO_2-DOM hydrodynamic diameters by comparison factor, given differing pH, DOM concentration, and DOM type.

Group	Mean
**Least square means for DOM Type:**	
**HA**	224.65
**Colloids**	244.65
**TPI**	280.35
**HPO**	261.5
Standard Error of LS mean = 8.351	
**Least square means for DOM Conc:**	
**Zero**	238.75
**Lowest**	258.438
**Low**	254.5
**High**	255.125
**Highest**	257.125
Standard Error of LS mean = 9.337	
**Least square means for pH:**	
**3**	287.45
**5**	234.2
**7**	246.4
**9**	243.1
Standard Error of LS mean = 8.351	

Table S5. TiO_2 ζ-potential in the absence and presence of DOM at various pH values. Error-values represent standard error.

Samples		pH 3	pH 5	pH 7	pH 9
		ζ-potential			
**TiO_2**		+29.2 (±0.9)	+11.6 (±2.5)	-20.2 (±1.1)	-27.1 (±1.2)
**TiO_2 with HA**	**0.5 mgC/L**	-30.8 (±1)	-27.2 (±3.6)	-38.9 (±1.5)	-34.7 (±1.3)
	**1.0 mgC/L**	-29.8 (±1.1)	-28 (±2.4)	-36.8 (±1.8)	-37.3 (±1.3)
	**1.5 mgC/L**	-33.2 (±1.5)	-28.5 (±2.6)	-37.2 (±1.6)	-39.3 (±1.7)
	**2.0 mgC/L**	-29.9 (±1.2)	-27.4 (±1.7)	-38.4 (±1.4)	-40.9 (±1.5)
**TiO_2 with Colloids**	**0.5 mgC/L**	+16.3 (±1)	-20.7 (±2.6)	-27.4 (±1.1)	-30.0 (±1.3)
	**1.0 mgC/L**	-10.3 (±1)	-21.2 (±1.8)	-27.5 (±1.1)	-29.9 (±1.4)
	**1.5 mgC/L**	-11.8 (±0.9)	-21.5 (±1.7)	-29.0 (±1.2)	-30.1 (±1.2)
	**2.0 mgC/L**	-10.7 (±0.9)	-26.6 (±2.8)	-28.8 (±1.2)	-29.6 (±)
**TiO_2 with TPI**	**2.5 mgC/L**	+7.2 (±1.2)	-7.8 (±1.7)	-18.5 (±1.8)	-20.3 (±2)
	**5.0 mgC/L**	-2.5 (±1.2)	-16.7 (±2.6)	-19.9 (±1.2)	-20.6 (±2.6)
	**7.5 mgC/L**	-3.3 (±1.3)	-18.6 (±2.3)	-20.4 (±1.5)	-20.8 (±1.7)
	**10 mgC/L**	-2.8 (±1.2)	-20.2 (±1.8)	-20.7 (±2)	-18.1 (±3.3)
**TiO_2 with HPO**	**2.5 mgC/L**	+24.8 (±2.3)	-1.8 (±2)	-16.6 (±1.8)	-16.6 (±1.8)
	**5.0 mgC/L**	+18.6 (±1.8)	-9.5 (±2.7)	-17.6 (±2.2)	-18.7 (±2)
	**7.5 mgC/L**	+11.8 (±1.9)	-13.2 (±2.6)	-18.4 (±2.2)	-17.6 (±1.4)
	**10 mgC/L**	+9.0 (±1.2)	-14.8 (±2.4)	-19.2 (±2.9)	-20.0 (±1.9)

Table S6. Three-factor ANOVA results for TiO_2 ζ-potential with changing pH, DOM type, and DOM concentration.

Source of Variation	DF	SS	MS	F	P
**DOM Type**	3	4821.071	1607.024	18.234	<0.001
**DOM Conc**	4	4254.697	1063.674	12.069	<0.001
**pH**	3	10456.06	3485.354	39.546	<0.001
**Residual**	69	6081.242	88.134	-	-
**Total**	79	25613.07	324.216	-	-

Table S7. Pairwise multiple comparison results for TiO_2 ζ-potential using the Holm-Šidák method, given differing pH, DOM concentration, and DOM type.

Comparison	Diff. of Means	t	P	P<0.050
**Comparison factor: DOM Type**				
**TPI vs. HA**	20.925	7.048	<0.001	Yes
**HPO vs. HA**	15.712	5.292	<0.001	Yes
**TPI vs. Colloids**	10.95	3.688	0.002	Yes
**Colloids vs. HA**	9.975	3.36	0.004	Yes
**TPI vs. HPO**	5.737	1.932	0.112	No
**HPO vs. Colloids**	5.213	1.756	0.084	No
**Comparison factor: DOM Concentration**				
**Zero vs. Highest**	19.571	5.896	<0.001	Yes
**Zero vs. High**	19.069	5.745	<0.001	Yes
**Zero vs. Low**	17.606	5.304	<0.001	Yes
**Zero vs. Lowest**	13.564	4.087	<0.001	Yes
**Lowest vs. Highest**	6.007	1.81	0.372	No
**Lowest vs. High**	5.506	1.659	0.415	No
**Lowest vs. Low**	4.042	1.218	0.644	No
**Low vs. Highest**	1.964	0.592	0.912	No
**Low vs. High**	1.463	0.441	0.885	No
**High vs. Highest**	0.501	0.151	0.88	No
**Comparison factor: pH**				
**3 vs. 5**	28.612	9.638	<0.001	Yes
**3 vs. 9**	26.772	9.018	<0.001	Yes
**3 vs. 7**	14.832	4.996	<0.001	Yes
**7 vs. 5**	13.78	4.642	<0.001	Yes
**9 vs. 5**	11.94	4.022	<0.001	Yes
**7 vs. 9**	1.84	0.62	0.537	No

Table S8. Least square means for TiO_2 ζ-potential by comparison factor, given differing pH, DOM concentration, and DOM type.

**Group**	**Mean**
**Least square means for DOM Type:**	
**HA**	-27.24
**Colloids**	-17.265
**TPI**	-11.528
**HPO**	-6.315
Standard Error of LS mean = 2.099	
**Least square means for DOM Conc:**	
**Zero**	-1.625
**Lowest**	-15.189
**Low**	-19.231
**High**	-20.694
**Highest**	-21.196
Standard Error of LS mean = 2.347	
**Least square means for pH:**	
**3**	1.967
**5**	-12.865
**7**	-24.805
**9**	-26.645
Standard Error of LS mean = 2.099	

Table S9. Average hydrodynamic diameters of TiO_2 particles in the absence and presence of 10 mgC/L TPI or HPO with different co-solute conditions. Error-values represent standard error.

Salt and Ionic Strengths (mM)		DI Water	10 mgC/L TPI	10 mgC/L HPO
		Hydrodynamic Diameter (nm)		
**NaCl**	**0**	210 (±20)	191 (±28)	234 (±40)
	**1**	268 (±30)	187 (±31)	202 (±26)
	**100**	323 (±53)	319 (±40)	323 (±46)
	**1000**	366 (±49)	337 (±41)	364 (±60)
**$CaCl_2$**	**3**	308 (±28)	287 (±40)	301 (±51)
	**300**	325 (±39)	330 (±31)	323 (±49)
	**3000**	410 (±54)	380 (±43)	592 (±78)
**$Al_2(SO_4)_3$**	**1.5**	210 (±25)	269 (±36)	314 (±45)
	**150**	327 (±30)	342 (±43)	353 (±40)
	**1500**	419 (±66)	443 (±95)	419 (±52)

Table S10. Three-factor ANOVA results for TiO_2-DOM particle hydrodynamic diameters with changing IS, ion species, and DOM Type.

Source of Variation	DF	SS	MS	F	P
**Salt Type**	2	14242.722	7121.361	4.44	0.021
**Ionic Strength**	3	209237.444	69745.815	43.489	<0.001
**DOM Type**	2	8052.389	4026.194	2.51	0.099
**Residual**	28	44905.333	1603.762	-	-
**Total**	35	276437.889	7898.225	-	-

Table S11. Pairwise multiple comparison results for TiO_2-DOM hydrodynamic diameters using the Holm-Šidák method, given differing IS, ion species, and DOM type.

Comparison	Diff. of Means	t	P	P<0.050
**Comparison factor: Ionic Species**				
**$CaCl_2$ vs. NaCl**	47.25	2.89	0.022	Yes
**$Al_2(SO_4)_3$ vs. NaCl**	33.917	2.075	0.092	No
**$CaCl_2$ vs. $Al_2(SO_4)_3$**	13.333	0.816	0.422	No
**Comparison factor: IS**				
**High vs. Zero**	202.778	10.741	<0.001	Yes
**High vs. Low**	153.778	8.146	<0.001	Yes
**Medium vs. Zero**	117.778	6.239	<0.001	Yes
**High vs. Medium**	85	4.503	<0.001	Yes
**Medium vs. Low**	68.778	3.643	0.002	Yes
**Low vs. Zero**	49	2.596	0.015	Yes

Table S12. Least square means for TiO_2-DOM hydrodynamic diameters by comparison factor, given differing IS, ion species, and DOM type.

Group	Mean
**Least square means for DOM Type:**	
**NaCl**	277
**$CaCl_2$**	324.25
**$Al_2(SO_4)_3$**	310.917
Standard Error of LS mean = 11.561	
**Least square means for DOM Conc:**	
**Zero**	211.667
**Low**	260.667
**Medium**	329.444
**High**	414.444
Standard Error of LS mean = 13.349	
**Least square means for pH:**	
**None**	298.833
**TPI**	288.917
**HPO**	324.417
Standard Error of LS mean = 11.561	

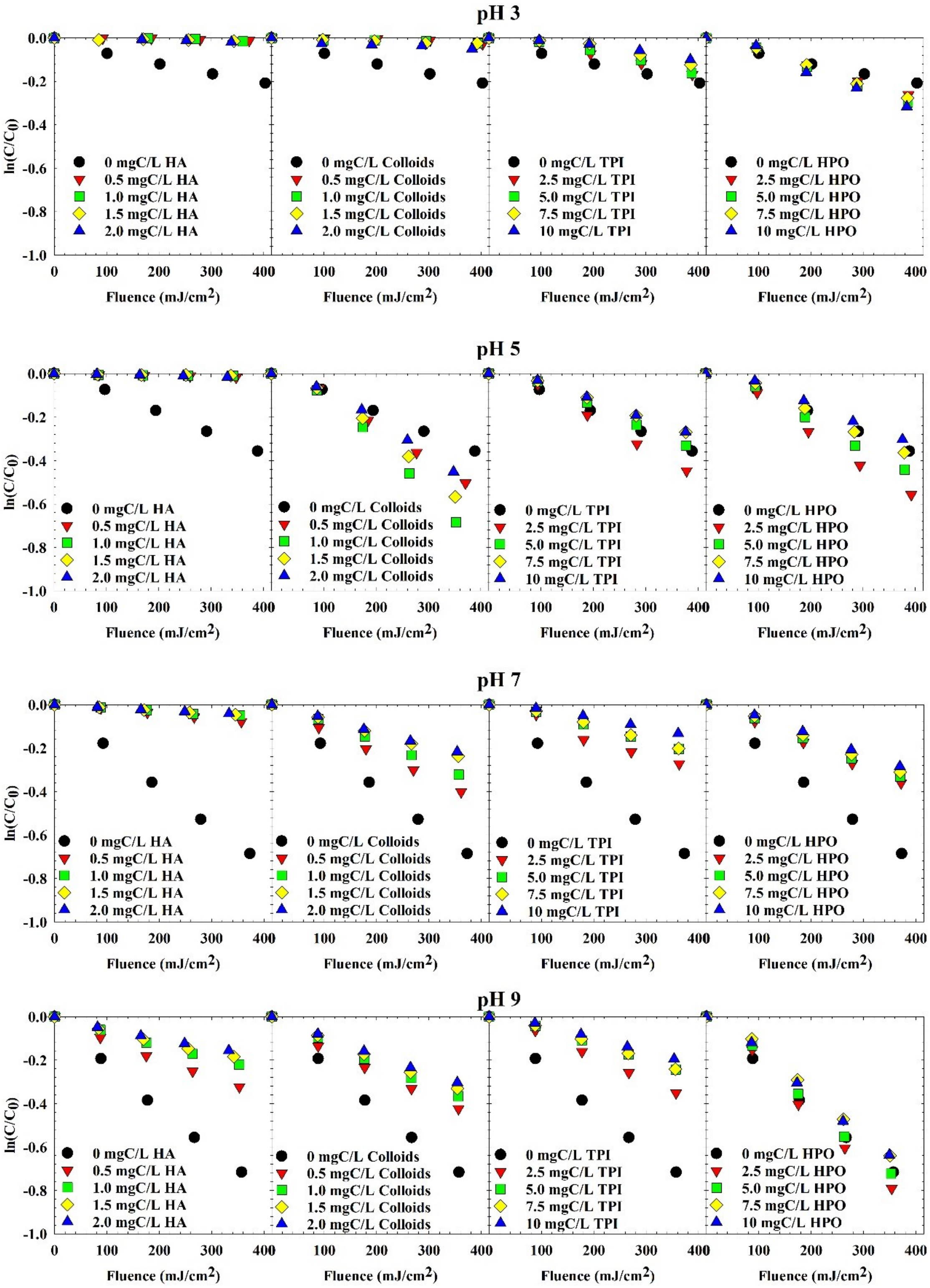

Figures S1. Photocatalytic degradation of *p*CBA as a function of fluence for different pH and DOM conditions (pH values vary by row and DOM type vary by column, with DOM concentrations varying within each panel).

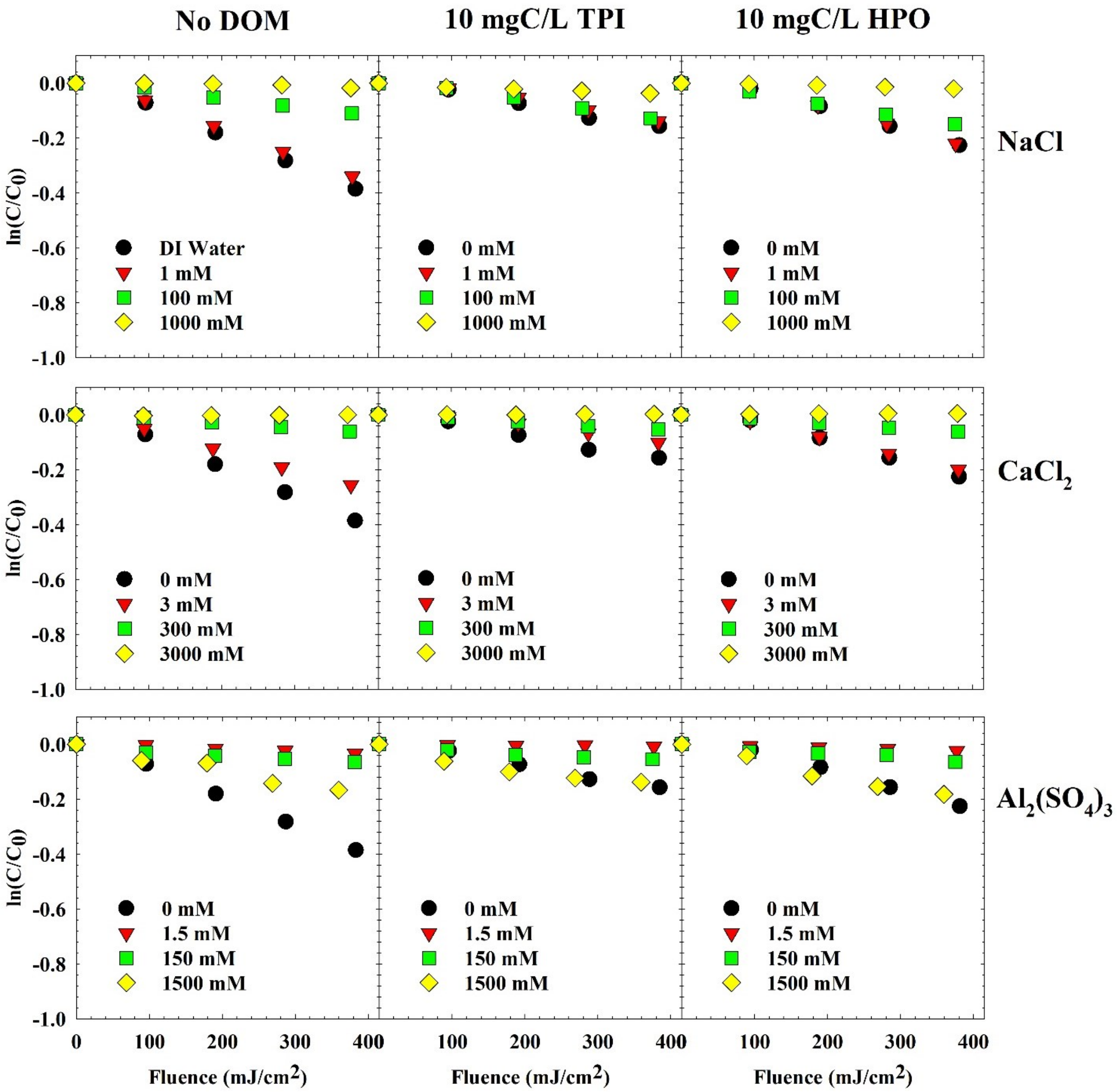

Figures S2. Photocatalytic degradation of *p*CBA as a function of fluence for different IS, ionic species, and DOM conditions (salt type vary by row and DOM conditions vary by column, with IS values varying within each panel).
